# Methylprednisolone and Hyaluronic Acid versus Each Agent Alone to Control Complication of Impacted Wisdom Removal

**DOI:** 10.1155/2022/1563513

**Published:** 2022-03-24

**Authors:** Alaa Abdelqader Altaweel, Abd El-Hamid Gaber, Mahmoud Zuhair Alnaffar, Elham Ahmed Alshomrani, Rahaf Asaad Alrehaili, Rahaf Abdulmoeen Alshaikh, Jumana Nader Baeshin, Elaf Sami Al-akhdar

**Affiliations:** ^1^Oral & Maxillofacial Surgery Department, Faculty of Dental Medicine for Boys, Al-Azhar University, Cairo, Egypt; ^2^Oral & Maxillofacial Surgery Department, Vision Colleges, Jeddah, Saudi Arabia; ^3^Clinical Pharmacology Department, Faculty of Medicine, Menoufia University, Shibin Al Kawm, Menoufia, Egypt; ^4^Maxillofacial Surgery Department, Vision Colleges, Jeddah, Saudi Arabia; ^5^Vision Colleges, Jeddah, Saudi Arabia

## Abstract

**Introduction:**

Extraction of impacted molars is commonest operation in oral cavity and associated with complications disturbing patient's quality of life. Hyaluronic acid is a nontoxic agent recommended for wound management due to its anti-inflammatory effects. Also, methylprednisolone sodium is used to reduce pain and edema. The aim this study was to compare the effect of combined use of corticosteroid and hyaluronic acid versus each agent alone in controlling postextraction complications of impacted mandibular third molars.

**Materials and Methods:**

This prospective randomized trial included patients suffering from impacted mandibular third molar. Patients were divided into four groups. Group I, control, and group II received methylprednisolone sodium succinate injection preoperatively, group III received hyaluronic acid applied in extraction socket, and group IV received preoperative methylprednisolone sodium succinate injection and topical hyaluronic acid in the socket. All patients were evaluated preoperatively and postoperative day to assess swelling, pain, mouth opening, and total analgesic dose.

**Results:**

Group IV showed insignificant edema along the study period, and other groups showed significant edema on third postoperative day that improved on seventh postoperative in group II and III and tenth postoperative day in group I. Significant decreased mouth opening occurred on second postoperative day in group I, while in other groups, it occurred in third postoperative day. Significant improvement occurred on seventh postoperative day in all groups except in group I which occurred on tenth postoperative day. There was less pain and analgesic dose reported in group IV than other groups.

**Conclusion:**

Combined use of methylprednisolone sodium succinate and hyaluronic acid significantly decrease postoperative complications than using each agent alone.

## 1. Introduction

In oral and maxillofacial surgery, surgical extraction of impacted third molars accounts for a considerable number of patients. It necessitates meticulous preoperative planning, the application of surgical skills during the procedure, and good postoperative care [1].

Pain, limited mandibular movement, and edema are all common side effects after removing an impacted lower third molar. Pain usually begins three hours after surgery and ranges in intensity from moderate to severe. Furthermore, impaction removal can cause considerable edema in the operational area, which is caused by phospholipids being converted to arachidonic acid by phospholipase A2, and formation of prostaglandins, leukotrienes, or thromboxane-related substance [2].

Corticosteroids have a variety of effects on the human body. The usual rate of hydrocortisone production is 15–30 mg per day, but at times of stress, it can reach 300 mg. Exogenous hydrocortisone must be given at doses that surpass the physiological limit of secreted hydrocortisone to reduce inflammation. Methylprednisolone sodium succinate is a synthetic corticosteroid that has a stronger anti-inflammatory effect. Methylprednisolone sodium succinate has a potency of nearly five times that of hydrocortisone [3]. Because it prevents phospholipid conversion to arachidonic acid, it has been utilized to reduce inflammatory sequelae following impaction removal [4].

Hyaluronic acid (HA) is a biocompatible, high-molecular glycosaminoglycan that is found in the connective tissue and synovial fluid. It regulates tissue hydration and mechanism of cell detachment [5, 6]. HA has been shown to conduct a wide range of functions in previous investigations. It, for example, has an anti-inflammatory impact during oral wound healing, supports tissue integrity in terms of osmotic pressure and tissue lubrication, and maintains joint synovial fluid viscosity [7–9]. Furthermore, because HA is nonimmunogenic and nontoxic, it can be used safely in medicine [10, 11]. In the wound healing process, HA serves a variety of functions. It was first used in dentistry to treat periodontal disease such as gingivitis. Clinically, local application has yielded positive outcomes. On the contrary, it has been utilized to reduce orally administered analgesic doses in the postoperative phase of knee surgery [12].

This study was designed to compare the effect of combined use of methylprednisolone sodium succinate and hyaluronic acid versus each agent alone in controlling postextraction complications of impacted mandibular third molars.

## 2. Materials and Methods

### 2.1. Patient Selection

This study was a prospective randomized controlled clinical trial. All patients included in the study provided a signed statement of informed consent. The study duration was from February 2021 till June 2021 and ended as planned in the suggested study's protocol. The study was performed according to rules of ethics declared by Helsinki, and ethical approval from the institutional ethics committee was obtained (21–5/2). This study was registered under https://www.ClinicalTrial.gov (study no. https://clinicaltrials.gov/ct2/show/NCT04816253-23\3\2021). The study was performed according to CONSORT 2010 (Consolidated Standards of Reporting Trials) and CONSORT 2010 flow diagram (Supplementary file (available here)).

Patients were included in this study if they were medically healthy, between the ages of 20 and 40, and had a mesioangular impacted mandibular third molar (Pell and Gregory class B, vertical position, and Pell and Gregory class 2, horizontal relation to ramus). Patients who had severe pericoronitis, concomitant carious and/or periodontal disease, or who had contraindications to the medicines or anesthetics utilized in the trial were excluded from the study.

### 2.2. Study Design

The minimum sample size required of subjects was 69 using G^*∗*^Power version 3.1.92, where the effect size was 0.40 with alpha error 0.05 and a power level of 0.80. Considering that some patients could be lost during follow-up postoperative periods, 72 subjects were finally included.

All of surgeries were performed by the same oral and maxillofacial surgeon and assistant. The patients were divided randomly, using online software (https://www. randomizer.org), into four groups each contained 18 patients: in group I, after extraction of impacted third molar, the wound was irrigated by normal saline before closure; in group II, methylprednisolone sodium succinate (Pfizer, Germany) was injected 125 mg single dose intravenously (IV) one hour before the surgery; in group III, 2 ml HA (Raiser Pharma, Italy) was placed in the socket after tooth extraction; while in group IV, methylprednisolone sodium succinate was injected 125 mg single dose IV one hour before the surgery and 2 ml HA was placed in the socket after tooth extraction before closure.

### 2.3. Surgical Protocol

**Figure 1:**
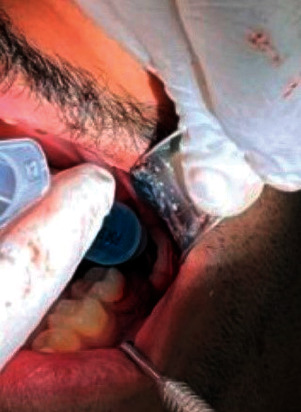
Application of HA after impaction removal.

All surgeries were performed under local anesthesia using 2% lidocaine with 1 : 200000 epinephrine solution to block the inferior alveolar nerve, lingual nerve, and buccal nerve. In groups II and IV, 125 mg methylprednisolone sodium succinate was injected IV one hour before surgery. Full thickness mucoperiosteal flap was elevated to gain access to the surgical area, buccal and distal bone around the impacted molar was removed using surgical bur rotate at low speed under copious irrigation, and tooth separation was carried out. After tooth extraction, bone edge was smoothed using bone file, and socket was irrigated with sterile normal saline. After that, 2 ml HA was applied in groups III and IV (Figure 1). Then, the flap was closed was with 3–0 silk. All patients received augmentin 1 g/12-hour, ibuprofen 400 mg on demand, and written postoperative instructions.

### 2.4. Patient Evaluation

Mandibular movement was assessed by measuring a distance between upper and lower anterior teeth, total amount of analgesic was calculated during the postoperative period, pain intensity was recorded on the visual analogue scale (VAS), the patient signs a mark on the scale to indicate a pain intensity range from no pain “0” to unbearable pain “10,” and facial swelling was evaluated by using a modified Gabka method [13]. The points of this measurement include tragus (T), soft tissue pogonion (P), ala of nose (AN), lateral canthus of eye (CE), mandible angle (MA), and mouth corner (MC). Seven different measurements (L1–L7) were recorded: L1, T-P; L2, T-MC; L3, T-AN; L4, MA-P; L5, MA-MC; L6, MA-AN; and L7, MA-CE.

Mouth opening, pain on VAS, and facial swelling measurements were taken preoperatively and on first, second, third, seventh, and tenth postoperative days.

### 2.5. Statistical Analysis

Statistical analysis was carried out using Statistical Package for Social Sciences program (SPSS) version 26.0 software (Chicago, IL, USA). For descriptive statistics, the means, standard deviations, and 95% confidence intervals were used for quantitative variables. For analytic statistics, a repeated measures ANOVA with a Greenhouse–Geisser correction test was applied to assess differences in means of quantitative variables within the same group at different time periods, while the Kruskal–Wallis test was used to assess differences in means of quantitative variables between the four groups at each time period. The statistical methods were verified, assuming a significant level of *P* < 0.05 and a highly significant level of *P*=0.001.

## 3. Results

This study was conducted on 72 patients, 38 females and 34 males, with overall mean age 26.05 ± 7.34. The mean patients' age was 26.41 ± 7.52, 27.11 ± 6.2, 27.40 ± 8.33, and 27.40 ± 7.31 for groups I, II, III, and IV, respectively. There was 10, 11, 9, and 8 female patients in groups I, II, III, and IV respectively.

Regarding age and sex distribution, there was no statistical difference among groups. Also, regarding all outcome variables at the preoperative period, there was no significant difference among groups. There was no evidence of infection; there was normal wound healing except one case in the HA group which showed wound dehiscence during third postoperative day that healed on seventh postoperative day using good oral hygiene measures. Also, there were 2 cases that showed paresthesia related to lingual nerve distribution in control and combined HA-methylprednisolone sodium succinate group.

Regarding inflammatory swelling (L1–L4), (L5–L7), and (L1–L7), this study showed that on 2^nd^ postoperative day, there was significant edema in group I (*P*=0.001), (L5–L7; *P*=0.034), (L1–L7; *P*=0.002) and group II (L1–L4; *P*=0.001), (L5–L7; *P*=0.004). On third postoperative day, it was significant in all groups; in group I (L1–L4, *P*=0.001, L5–L7, *P*=0.009, L1–L7, *P*=0.001), in group II (L1–L4, *P*=0.001, L5–L7, *P*=0.001), and in group III (L1–L4, *P*=0.003, L5–L7, *P*=0.001, L1–L7, *P*=0.001) except group IV which showed insignificant edema throughout the study (*P* < 0.05).

A significant improvement appeared in seventh postoperative day in group II (L1–L4; *P*=0.003) and group III (L1–L4; *P*=0.016) and in group II (L5–L7, *P*=0.009) and group III (L1–L7, *P*=0.005), while significant improvement appeared in tenth postoperative day in group I (L5–L7, *P*=0.021 and L1–L7, *P*=0.001) and group III (L5–L7, *P*=0.005). From first to seventh postoperative days (L1–L4), there was significant edema in group I than group IV (*P* < 0.05 in all) that means significant improvement in group IV than group I (Table 1).

**Figure 2:**
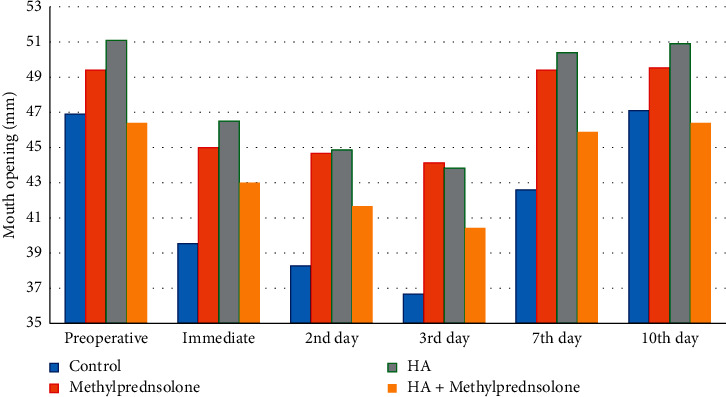
Mouth opening in all groups at different follow-up intervals.

Significant decrease in mouth opening occurred on second postoperative day in group I (*P* < 0.005), while in groups II (*P* < 0.020), III (*P* < 0.025), and IV (*P* < 0.021), significant decrease occurred on third postoperative day. Significant improvement occurred in seventh postoperative day as compared with third postoperative day in all groups (where in group I (*P* < 0.019), group II (*P* < 0.011), and in group IV (*P* < 0.022)), except group I occurred at tenth postoperative day (*P* < 0.001). On third postoperative day, there was a significant decrease in mouth opening in group I than groups II (*P* < 0.002) and IV (*P* < 0.046). On seventh postoperative day, there was significant improvement in groups II (*P* < 0.001), III (*P* < 0.001), and IV (*P* < 0.002) than in group I (Figure 2).

**Figure 3:**
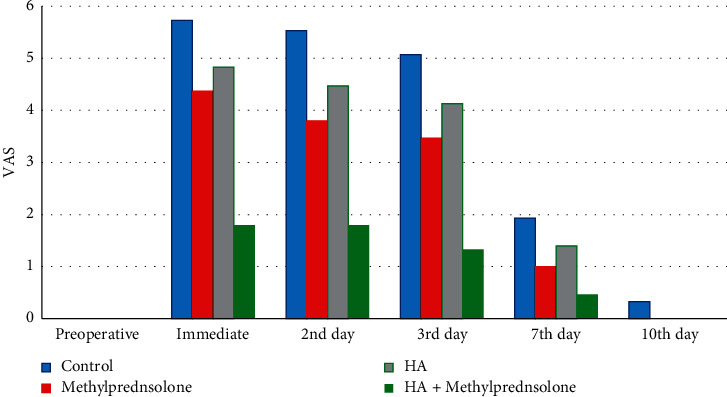
Pain level on VAS in all groups along different study periods.

Regarding pain level on VAS, this study showed that the pain appeared in all groups immediately postoperative and continued to third postoperative day which significantly decreased on seventh postoperative days in all groups (in groups I, II, and III (*P* < 0.001) and in group IV (*P* < 0.001)). Comparison among groups showed that in all postoperative periods, the pain was significantly minimum in group IV than other groups (*P* < 0.001 except at tenth postoperative day where there was no pain reported in group IV) and its maximum degree was in group I. The pain was significantly less in group II than group I in all postoperative days (*P* < 0.001 from first to seventh postoperative day, while no pain reported at tenth postoperative day in group II) and was less in group III than group I on first (*P*=0.014), second (*P* < 0.001), and third postoperative days (*P* < 0.002) (Figure 3).

**Figure 4:**
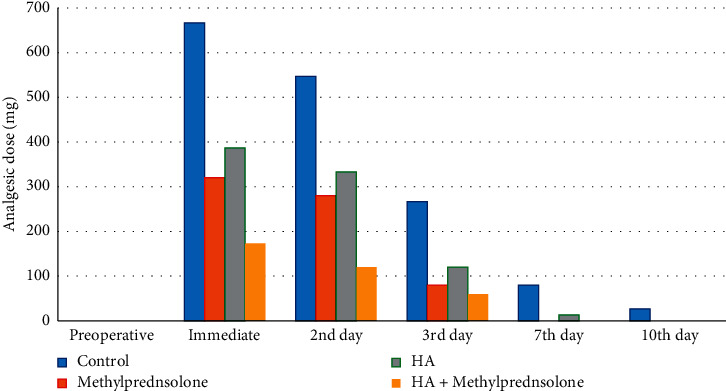
Analgesic dose in all groups along different study periods.

Significant reduction in analgesic dose occurred on second postoperative day in all groups (where group II (*P*=0.043), group III (*P*=0.007), group IV (*P*=0.013)), except group I (*P*=0.133), where significant reduction occurred on third postoperative day (*P* < 0.001). Comparison among groups showed that during first two days postoperative, less analgesic dose was reported in group IV than other groups (*P* < 0.001), and there was significantly less analgesic dose in groups II (*P* < 0.001) and III (*P* < 0.002) than group I. During seventh postoperative day, there was less analgesic dose in group III than group I (*P* ≤ 0.018). At tenth postoperative day, there was more analgesic dose in group I (26.67 ± 70.37) than all other groups where no analgesic dose was not reported (Figure 4).

## 4. Discussion

Patients' quality of life deteriorates within the first week after extraction of impacted mandibular third molars [14, 15]. Corticosteroids have been used to control postoperative complication with good results [16, 17].

In the current study, methylprednisolone sodium succinate was used to control postoperative complications as it is five times as powerful as hydrocortisone, has no mineralocorticoid activity, and has a biologic half-life of 18–36 hours [18]. Also, IV rout of administration was used as it provides stable blood levels just prior to surgical trauma. While, oral route's effectiveness is dependent on patient compliance, and a repeated dose is necessary to maintain an adequate blood level. Ability of oral glucocorticoid to reduce postoperative sequelae is debatable. However, IM route has a longer anti-inflammatory effect, but it also has a larger risk of adrenal suppression [18].

A single preoperative dose of 125 mg methylprednisolone sodium succinate was used. As it was reported, this dose is associated with significant reduction of edema after third molar surgery and was not associated with adverse effects [19, 20]. Furthermore, doses higher than 125 mg methylprednisolone sodium succinate may cause a decrease in plasma cortisol levels for more than 48 hours [21].

Beside corticosteroid, different materials with different application techniques were applied to reduce complication associated with extraction of impacted mandibular third molars. Hyaluronic acid has been used in many fields to improve wound healing and pain relief without side effects [7, 22, 23]. Hanci and Altun reported on its application posttonsillectomy for pain relief [24].

To reach therapeutic approach for controlling postoperative complications associated with surgical removal of impacted mandibular third molar, the efficacy of the combined use of preoperative methylprednisolone sodium succinate injection and topical application of hyaluronic acid in the wound after extraction versus methylprednisolone sodium succinate injection or topical application of hyaluronic acid was evaluated.

In the current study, the impacted molar was removed using the same surgical technique through modified ward incision as it was concluded that duration of surgery and postoperative pain were significantly less in modified ward's flaps when compared with ward flap [25].

In the current study, there was no statistical difference among groups related to age and sex distribution. Also, there was no significant difference in all parameters during the preoperative period in all groups. This ensures more consistent results.

Results of this study showed that insignificant edema developed in group IV. Comparing all groups demonstrated that there was rapid relief of edema in methylprednisolone sodium succinate and HA groups at seventh postsurgical day than the control group that was achieved at tenth postsurgical. This result is in agreement with Bayoum et al. [26] who reported that topical application of HA gel in extraction site reduced facial swelling on seventh postoperative day and Koray et al. [7] who evaluated efficacy of HA spray after third molar extraction and reported reduction of swelling. Also, in agreement with Raakesh et al. [16] and Esen et al. [27], the former reported significant improvement in postoperative edema in dexamethasone than the control group and the later found significant decreased edema with the use of methylprednisolone sodium succinate than the control [28].

This is due to the hydrophilic nature of HA gel, which rapidly absorbs fluid at the surgical site, peaking on the third postoperative day and subsiding on the seventh. In addition, by scavenging reactive oxygen species such superoxide radicals (O_2_^−−^) and hydroxyl radicals (OH) and blocking inflammatory cell-derived serine proteinases, HA plays an important anti-inflammatory role through the inhibition of tissue degradation and speed up healing. Finally, HA's antiedematous properties could be linked to its osmotic buffering ability [29–31]. The ability of methylprednisolone sodium succinate to reduce inflammatory edema could be due to its ability to prevent phospholipid conversion to arachidonic acid, thus blocking the synthesis of other products such as prostaglandins, leukotrienes, and substances related to thromboxane A2, as well as its ability to reduce capillary permeability [32].

Regarding mouth opening, this study showed that there was significant decreased mandibular movement in the control group than methylprednisolone sodium succinate and combined methylprednisolone sodium succinate—HA groups on third postoperative day. On seventh postoperative day, there was significant improvement in mandibular movement in all groups than the control. This result is in agreement with Raakesh et al. [16], Elhag et al. [33], and Graziani et al. [34] who reported significant reduction in trismus in dexamethasone than the control group. This is also in agreement with Esen et al. [27] who found significant improved mouth opening with the use of methylprednisolone sodium succinate.

Most surgical procedures cause some edema or swelling, which can contribute to trismus [35]. Methylprednisolone sodium succinate [27, 32] and HA [29–31] have anti-inflammatory properties that help to reduce edema and thereby improve function.

The pain from removing an impacted third molar peaks within 4 hours of surgery, and most patients require analgesic medication [17]. This study demonstrated that after significant increased pain and analgesic dose on the immediate postoperative period, there was a significant reduction in pain on 2^nd^ postoperative day in combined methylprednisolone sodium succinate, HA group, on third postoperative day in methylprednisolone sodium succinate and HA groups, and on seventh postoperative day in the control group. Also, there was significant reduction in analgesic dose occurred in 2^nd^ postoperative day in all groups except the control on third postoperative day. Comparison among groups showed that during first 2 days postoperative, less analgesic dose was reported in group IV than other groups and there was significantly less analgesic dose in groups II and III than in group I.

These results are in accordance with Esen et al. [27] who found significantly less pain with the use of methylprednisolone sodium succinate than the control group and Koray et al. [7], Bayoum et al. [26], and Yilmaz et al. [36] who reported significantly less pain in hyaluronic acid than the control group.

The capacity of HA to inhibit bradykinin receptors is thought to be responsible for its analgesic action. Furthermore, it was claimed that the molecular weight of HA and its analgesic properties are related [37]. Furthermore, the anti-inflammatory properties of methylprednisolone sodium succinate and HA account for their function in lowering pain and analgesic dose following surgery [34].

In this investigation, IV injection of methylprednisolone sodium succinate had no adverse effects. This result is in agreement with Esen et al. [27] and could be attributable to the fact that single dose of methylprednisolone sodium succinate had no effect on adrenal function [38]. Although no side effects have been recorded, clinicians should be mindful of the likelihood of an allergic reaction to a corticosteroid or its vehicle, especially in patients with asthma.

The anti-inflammatory, antiedematous, and analgesic effects of methylprednisolone sodium succinate and HA interpret the better results achieved in the combined methylprednisolone sodium succinate, HA group than other groups.

One of the limitations of the present study is the absence of three-dimensional evaluation of facial edema. So, it is recommended to plan a future study with evaluation of swelling using soft tissue images obtained by the three-dimensional system that can provide a more reliable evaluation in that regard.

## 5. Conclusion

This study concluded that the combined use of methylprednisolone sodium succinate and hyaluronic acid significantly decreases postoperative edema, pain, and trismus than using methylprednisolone sodium succinate or hyaluronic acid alone.

## Figures and Tables

**Table 1:** Inflammatory swelling in all groups at different study periods.

Variables	Preoperative	Postoperative					*P* value
		1^st^ day	2^nd^ day	3^rd^ day	7^th^ day	10^th^ day	
Mean L1–L4							
Group I	9.87 ± 0.82	10.61 ± 0.77	10.83 ± 0.74	11.11 ± 0.78	10.95 ± 0.8	9.92 ± 0.76	<0.001^*∗*^
Group II	9.86 ± 0.4	10.01 ± 0.68	10.39 ± 0.47	10.45 ± 0.41	9.8 ± 0.76	9.8 ± 0.78	0.68
Group III	9.55 ± 0.93	10.28 ± 1.01	10.31 ± 0.8	10.95 ± 1.59	9.83 ± 0.98	9.65 ± 0.47	0.007^*∗*^
Group IV	9.96 ± 0.62	9.81 ± 1.27	9.83 ± 0.69	10.02 ± 0.81	9.71 ± 0.52	9.95 ± 0.73	0.96
*P* value	0.149	0.039^*∗*^	<0.001^*∗*^	<0.001^*∗*^	0.043^*∗*^	0.68	

Mean L5–L7							
Group I	11.82 ± 1.64	12.6 ± 1.71	13.03 ± 1.66	13.29 ± 1.56	12.51 ± 1.66	11.98 ± 1.69	0.034^*∗*^
Group II	11.58 ± 1.01	11.98 ± 1.08	12.65 ± 1.06	12.8 ± 1.02	11.84 ± 1.07	11.6 ± 1.16	0.015^*∗*^
Group III	12.03 ± 0.69	12.28 ± 0.82	12.43 ± 0.77	12.91 ± 0.76	12.14 ± 0.72	12.08 ± 0.88	0.020^*∗*^
Group IV	12.55 ± 0.67	12.91 ± 0.69	13.01 ± 0.7	13.08 ± 0.62	12.81 ± 0.61	12.47 ± 0.7	0.079
*P* value	0.092	0.22	0.32	0.41	0.07	0.27	

Mean L1–L7							
Group I	10.76 ± 1.14	11.72 ± 1.14	12.04 ± 1.11	12.16 ± 1.09	10.96 ± 1.12	10.81 ± 1.06	<0.001^*∗*^
Group II	10.9 ± 0.61	11.23 ± 0.72	11.3 ± 0.64	11.26 ± 0.64	11.04 ± 0.83	10.93 ± 0.73	0.389
Group III	10.61 ± 0.71	11.14 ± 0.85	11.22 ± 0.65	11.66 ± 1.04	10.73 ± 0.82	10.69 ± 0.57	0.007^*∗*^
Group IV	10.96 ± 0.55	11.14 ± 0.91	11.19 ± 0.59	11.31 ± 0.6	11.04 ± 0.53	11.03 ± 0.66	0.70
*P* value	0.31	0.59	0.14	0.045^*∗*^	0.71	0.28	

^*∗*^Significance: *P* value in column indicates significance at different follow-up periods in each group, while in row indicates significance between groups.

## Data Availability

The data used to support the findings of this study are available from the corresponding author upon request.

## Supplementary Materials

Supplementary Materials The study was performed according to CONSORT 2010 (Consolidated Standards of Reporting Trials) and CONSORT 2010 flow diagram.
