# Supplementary material for: Methylprednisolone and Hyaluronic Acid versus Each Agent Alone to Control Complication of Impacted Wisdom Removal
Source: Evid Based Complement Alternat Med. 2022 Mar 24;2022:1563513. doi: 10.1155/2022/1563513 (PMC8970876; doi:10.1155/2022/1563513)
Supplement: Supplementary Materials — The study was performed according to CONSORT 2010 (Consolidated Standards of Reporting Trials) and CONSORT 2010 flow diagram. [file 1563513.f1.zip › 1563513.f1/CONSORT Flow Diagram (1).doc]

**CONSORT 2010**

**Allocation**

**Analysis**

**Follow-Up**

**Enrollment**

Assessed for eligibility (n= 79)

Excluded (n= 7)

♦  Not meeting inclusion criteria (n= 5)

♦  Declined to participate (n=2)

♦  Other reasons (n=0)

Analysed (n= 18 )
♦ Excluded from analysis (give reasons) (n=0)

Lost to follow-up (give reasons) (n= 0 )

Discontinued intervention (give reasons) (n= 0)

Allocated to intervention (n= 18)

♦ Received allocated intervention (n=18 )

♦ Did not receive allocatedreasons) (n= 0 )

Lost to follow-up (give reasons) (n= 0 )

Discontinued intervention (give reasons) (n= 0)

Allocated to intervention

(n= 18)

♦ Received allocated intervention (n=18 )

♦ Did not receive allocatedreasons) (n= 0 )

Analysed (n= 18 )
♦ Excluded from analysis (give reasons) (n=0)

Randomized (n= 72)

Allocated to intervention (n= 18)

♦ Received allocated intervention (n=18 )

♦ Did not receive allocatedreasons) (n= 0 )

Allocated to intervention (n= 18)

♦ Received allocated intervention (n=18 )

♦ Did not receive allocatedreasons) (n= 0 )

Lost to follow-up (give reasons) (n= 0 )

Discontinued intervention (give reasons) (n= 0)

Lost to follow-up (give reasons) (n= 0 )

Discontinued intervention (give reasons) (n= 0)

Analysed (n= 18 )
♦ Excluded from analysis (give reasons) (n=0)

Analysed (n= 18 )
♦ Excluded from analysis (give reasons) (n=0)
